# Supplementary material for: Sen1 has unique structural features grafted on the architecture of the Upf1‐like helicase family
Source: EMBO J. 2017 Apr 13;36(11):1590–604. doi: 10.15252/embj.201696174 (PMC5452015; doi:10.15252/embj.201696174)
Supplement: Supplementary file 1 — Appendix [file EMBJ-36-1590-s001.pdf]

Appendix:

Sen1 has unique structural features grafted on the architecture of the Upf1-like helicase family

Supplementary information:

Figure S1

Table S1

Supplementary Methods

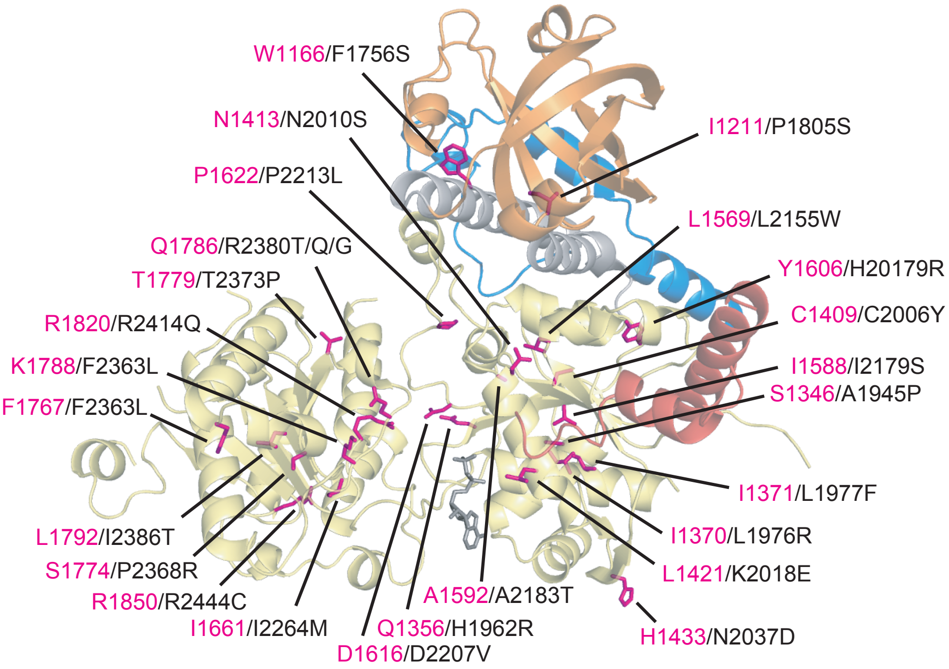

Figure S1 - AOA2-associated missense mutations.

Conserved AOA2-associated mutations mapped on the $Sen1_{Hel}$ structure. The residues that represent SETX missense mutations (black labels) are shown as sticks in magenta.

Table S1: List of oligonucleotides used in this work.

Name	Sequence (5'-3')	Information/use
DL1119	AAGTGACGAAGTTCATGCTA	Forward oligo to generate by PCR a probe to detect snR13 read-through region.
DL1154	CCTATAACAACAACAACATG	Forward oligo to generate by PCR a probe to detect snR47 RNA.
DL1157	ATAGCCATTAGTAAGTACGC	Reverse oligo to generate by PCR a probe to detect snR47 RNA.
DL1367	GGCCCAACAGTATATTCATATCC	Reverse oligo to generate by PCR a probe to detect snR13 read-through region.
DL2492	UGCAUUUCGACCAGGC	16-mer RNA oligonucleotide used for promoter-independent assembly of ECs. 5'-end FAM labeled.
DL2661	TAATACGACTCACTATAGGGGACCG GATGAGCGGTATTGAGTTTGAATTT ATCGATGGTATCAGATCTGGATCCT CGAGAAGCTGCGGGTACC	Non-template strand to produce by *in vitro* transcription the 75-mer RNA component of RNA:DNA duplexes used in unwinding assays in figure 6.
DL2662	GGTACCCGCAGCTTCTCGAGGATCC AGATCTGATACCATCGATAAATTCA AACTCAATACCGCTCATCCGGTCCC CTATAGTGAGTCGTATTA	Template strand to produce by *in vitro* transcription the 75-mer RNA component of RNA:DNA duplexes used in unwinding assays in figure 6.
DL3316	UUCAUUUCAGACCAGCACCCACUC ACUACAACUCACGACCAGGC	44-mer RNA oligonucleotide used to form the RNA:DNA duplexes used in unwinding assays in figures 1, 5 and EV1.
DL3352	CTAGAGGAAACAAACTATAGGAAA CGACCAGGCCCTCAACATCTCTCAC CCATCTCCACACGGGGGTTACCCGG CCTGCA	Non-template strand used in IVTT assays. Labeled with biotin at the 5'-end.
DL3353	GGCCGGGTAACCCCCGTGTGGAGAT GGGTGAGAGATGTTGAGGGCCTGGT CGTTTCCTATAGTTTGTTTCCT	Template strand used in IVTT assays. Labeled with biotin at the 5'-end.
DL3522	GGTACCCGCAGCTTCTCGAG	Short oligonucleotide for forming the RNA:DNA duplex containing a 5' single-strand overhang used in unwinding assays in figure 6.
DL3791	GCCTGGTCGTGAGTTGTAG	Short oligonucleotide that annealed to DL3316 forms the RNA:DNA duplex used in unwinding assays in figures 1 and 6. 5'-end FAM labeled.
DL3810	GCCTGGTCGTGAGTTGTAG	Same sequence as DL3791 but without the FAM label. Used as a competitor to prevent reannealing of the unwound oligo.
DL3841	GGTGCTGGTCTGAAATGAA	Short oligonucleotide that annealed to DL3316 forms an RNA:DNA duplex containing a 3' overhang used in unwinding assays in figure EV1. 5'-end FAM labeled.
DL3842	GGTGCTGGTCTGAAATGAA	Same sequence as DL3841 but without the FAM label. Used as a competitor to prevent reannealing of the unwound oligo.
ARE	UUUCUAUUUAUUUUG	15-mer RNA oligonucleotide used for fluorescence anisotropy measurements. 5'-end FL labeled.

Supplementary Methods:

Electrophoretic mobility shift assay

Assays were performed in 10 µl-reactions containing 10 mM Tris-HCl pH 7.5, 70 mM NaCl, 2 mM $MgCl_2$, 7.5 µM $ZnCl_2$, 10 µg/ml BSA, 10% glycerol and 1 mM DTT. The RNA substrate (2 nM final concentration) was incubated with increasing concentrations of $Sen1_{Hel}$ variants at 28°C for 15 min. Reactions were loaded on 6% native polyacrylamide gels and subjected to electrophoresis in 0.5XTBE at 100V for 80 min. Gels were scanned directly using a Typhoon scanner (GE Healthcare).

RNA expression analyses

RNAs were prepared using standard methods. Samples were separated by electrophoresis on 1.2% agarose gels, and then transferred to nitrocellulose membranes and crosslinked. Radiolabeled probes were prepared by random priming of PCR products covering the regions of interest with Megaprime kit (GE Healthcare) in the presence of α-^{32}P dCTP (3000 Ci/mmol). Oligonucleotides used to generate the PCR probes are listed in the Appendix Table S1. Hybridizations were performed using a commercial buffer (Ultrahyb, Ambion) and after washes, membranes were analysed by phosphorimaging.
