# Supplementary material for: Sen1 has unique structural features grafted on the architecture of the Upf1‐like helicase family
Source: EMBO J. 2017 Apr 13;36(11):1590–604. doi: 10.15252/embj.201696174 (PMC5452015; doi:10.15252/embj.201696174)
Supplement: Supplementary file 2 — Expanded View Figures PDF [file EMBJ-36-1590-s002.pdf]

Expanded View Figures

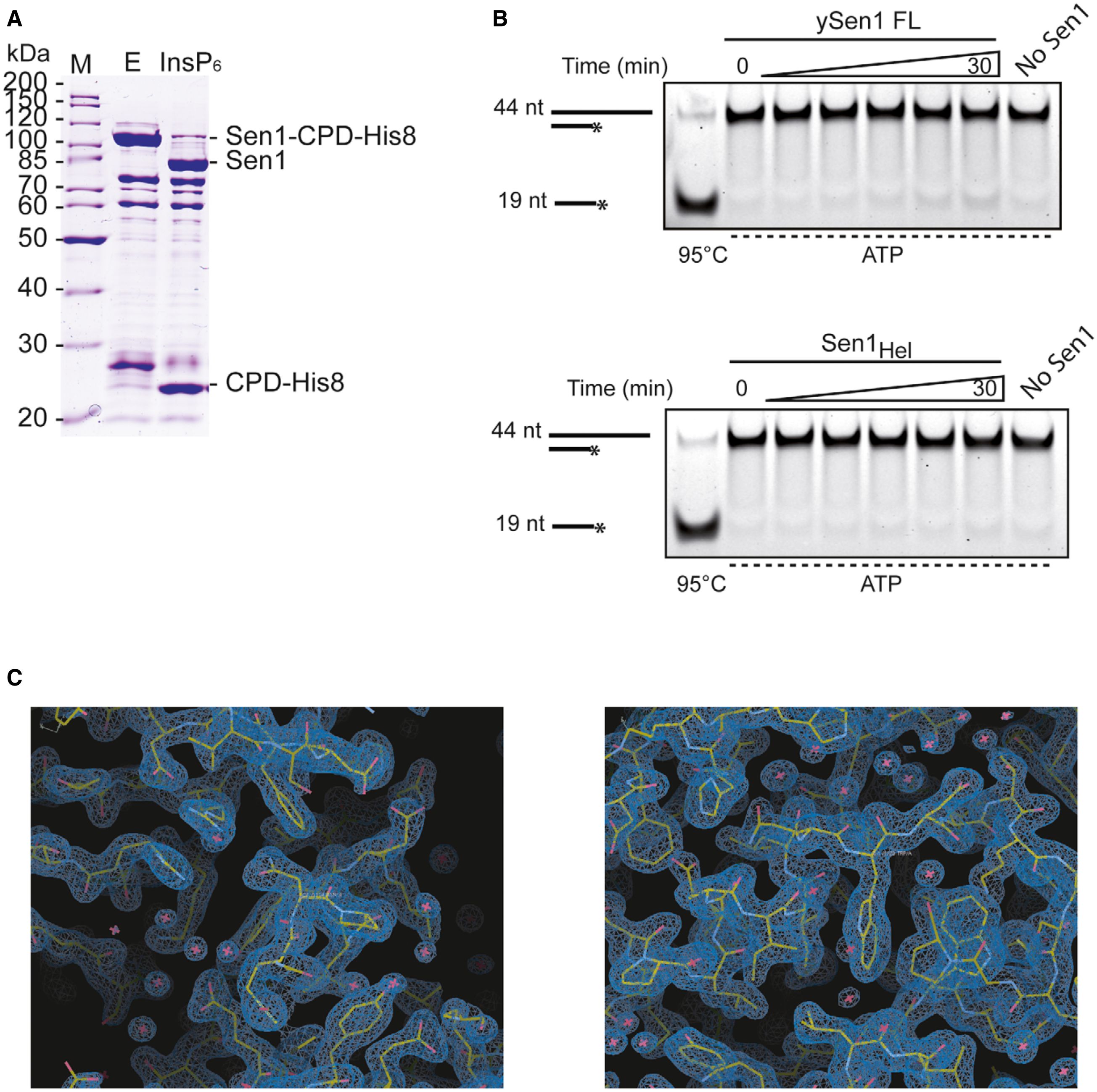

Figure EV1. Identification and characterization of a recombinant helicase core of *Saccharomyces cerevisiae* Sen1 suitable for structural studies.

A SDS–PAGE analysis of $Sen1_{976\text{-}1880}$ tagged with C-terminal CPD-His_8. Lane E shows the elution fraction after Ni^{2+}-affinity purification step, and lane InsP6 shows the tag cleavage after the protein was incubated with 400 μM inositol hexakisphosphate (InsP6) for 20 min at 4°C. The protein before and after tag cleavage is smaller than expected: Theoretical molecular weights of $Sen1_{976\text{-}1880}$-CPD-His_8 and $Sen1_{976\text{-}1880}$ are ~126 kDa and ~102 kDa, respectively. Left lane M shows a molecular weight marker.

B Time course analysis of the ATP-dependent 3′-5′ duplex unwinding activity of Sen1 proteins. Reactions were performed in the presence of 5 nM of Sen1 and 2 nM of substrate. An RNA:DNA duplex composed of a 44-mer RNA annealed to a 19-mer DNA molecule to provide a 3′-end 25-nt single-strand overhang was used as the substrate (see Appendix Table S1 for sequence details). The asterisk (*) denotes the presence of a FAM at the 5′ end of the DNA.

C Snapshots of the electron density maps at important regions of the structure described in the text. The 2Fo-Fc maps are contoured at 1.7σ.

 © 2017 The Authors

Figure EV2. Biochemical and structural properties of Sen1, and comparison with Upf1.

A Zoom-in view of the nucleotide binding site in Sen1 (left) and Upf1 (right) (PDB: 2XZO, Chakrabarti *et al*, 2011). The adenine ring is sandwiched between an apolar surface of RecA1 and an aromatic residue protruding from the short linker that connects RecA1 to RecA2 (Tyr1655, corresponding to $Tyr638_{Upf1}$ and $Tyr442_{IMGMBP2}$). In addition, the conserved side chain of Gln1339 (corresponding to $Gln413_{Upf1}$ and $Gln196_{IMGMBP2}$) forms a bidentate hydrogen-bond interaction with the N6 and N7 moieties of the adenine ring.

B Comparison of the structures of yeast $Sen1_{Hel}$-ADP, human $UPF1_{Hel}$-AMPPNPP (PDB: 2GJK, Cheng *et al*, 2007), $UPF1_{Hel}$-ADP:AlF_4^--RNA (PDB: 2XZO, Chakrabarti *et al*, 2011), and yeast $Upf1_{Hel\text{-}CH}$-ADP:AlF_4^--RNA (PDB: 2XZL, Chakrabarti *et al*, 2011). The molecules in a side-view orientation (90° clockwise rotation around a vertical axis with respect to the front-view in Fig 4A).

C Comparison of the RNA-binding sites of Sen1 (left) and Upf1 (right) (PDB: 2XZO, Chakrabarti *et al*, 2011).

D–F Functional analysis of the $Sen1_{Hel}$ T1289A, R1293A mutant harboring substitutions at conserved positions at the predicted RNA-binding surface. (D) Fluorescence anisotropy assays. Curves represent three independent measurements. (E) ATP hydrolysis assays. Values correspond to the average and SD of three independent experiments. (F) IVTT assays performed in the same conditions as in Fig 1C. The images correspond to different gels migrated and processed in parallel. The values of nascent RNA released correspond to one out of two independent experiments.

© 2017 The Authors

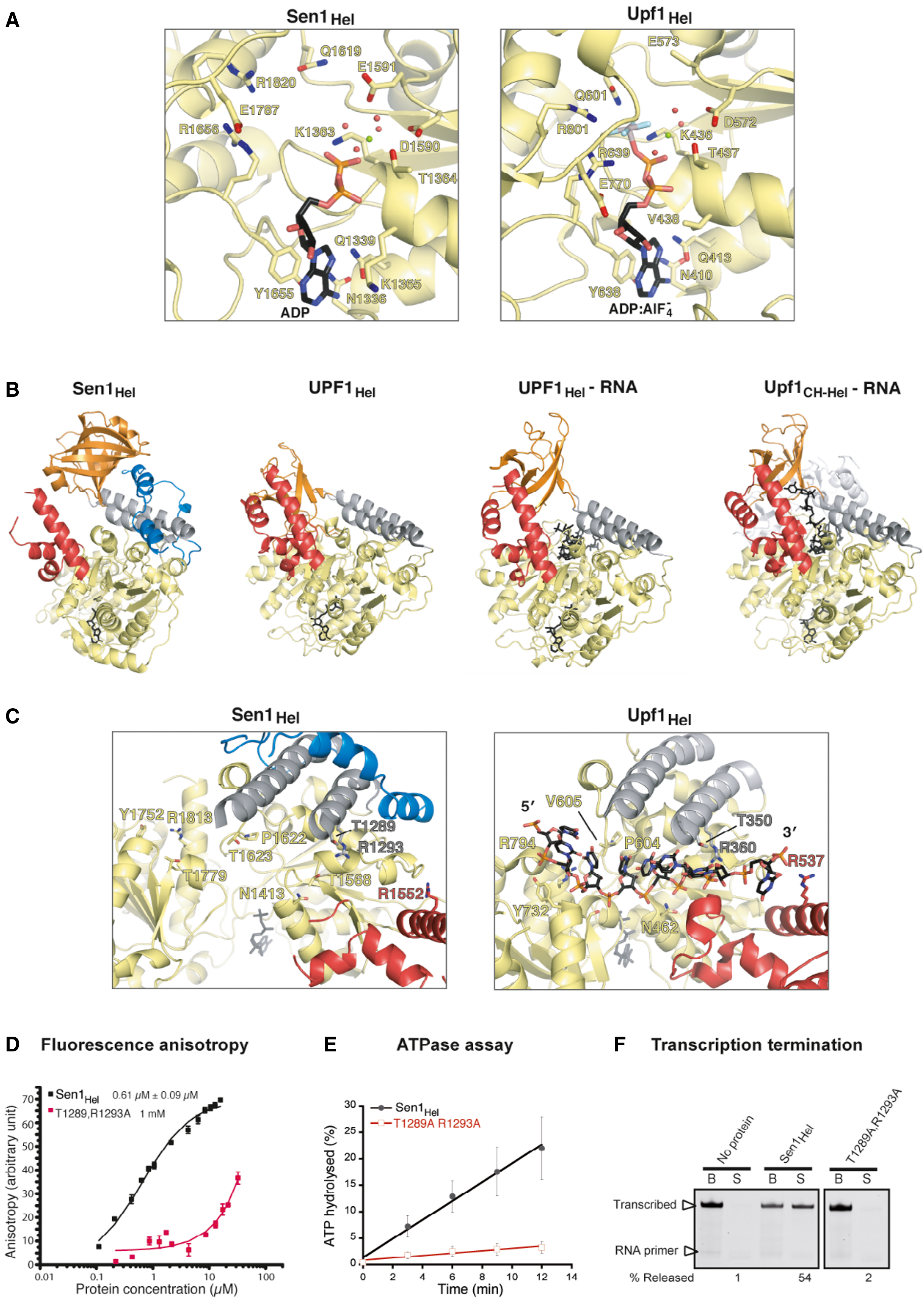

Figure EV2.

 © 2017 The Authors

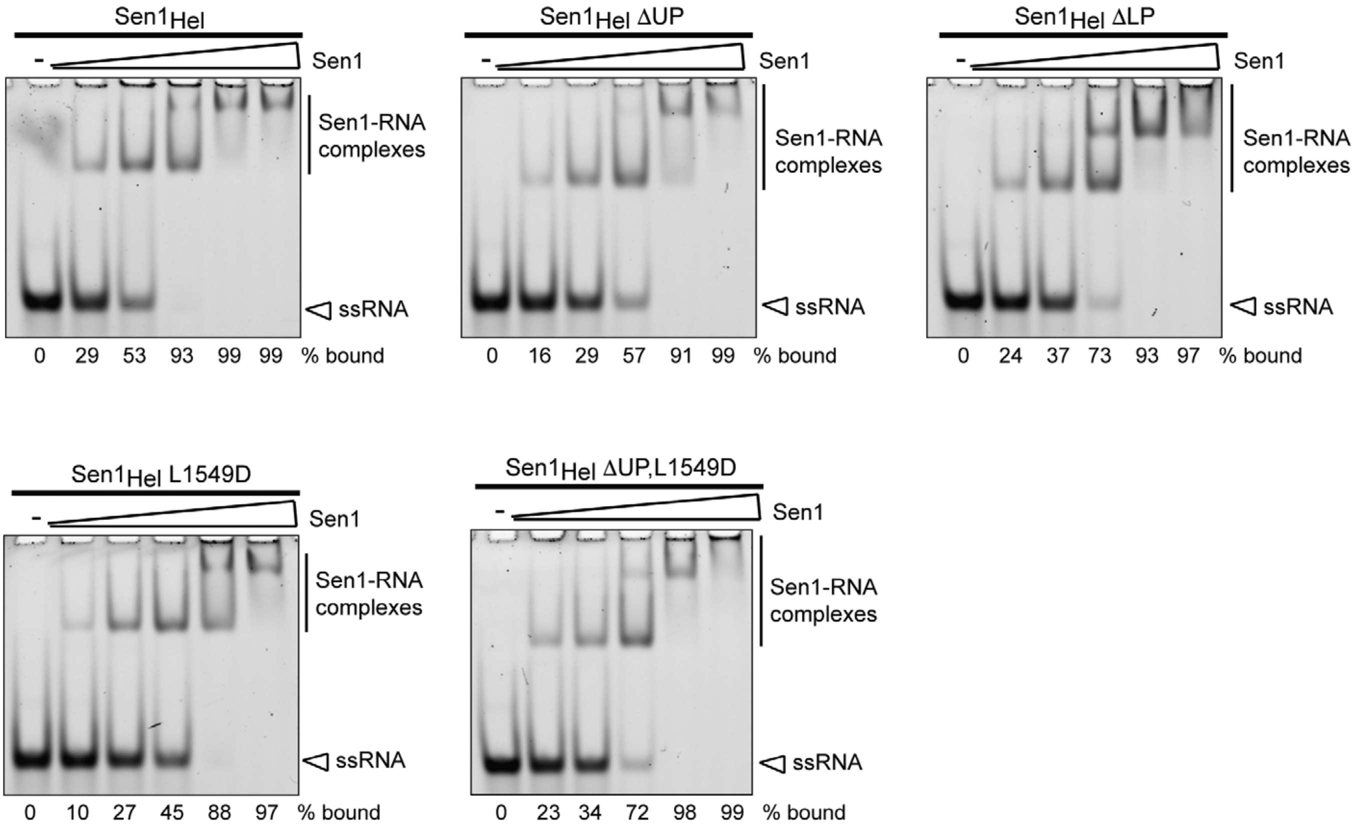

Figure EV3. Analysis of the impact of the "prong" mutations on the affinity of $Sen1_{Hel}$ for the RNA.
Electrophoretic mobility shift assay (EMSA) using a 5′-end fluorescently labeled 44-mer RNA as the substrate (DL3316, see Appendix Table S1) at 2 nM and $Sen1_{Hel}$ variants at 10, 20, 40, 80, and 160 nM at the final concentrations. Gels were migrated and processed in parallel. The values correspond to the mean of two independent experiments. At high protein concentrations, $Sen1_{Hel}$ forms high-order complexes with the RNA that are retained in the wells of the gel.

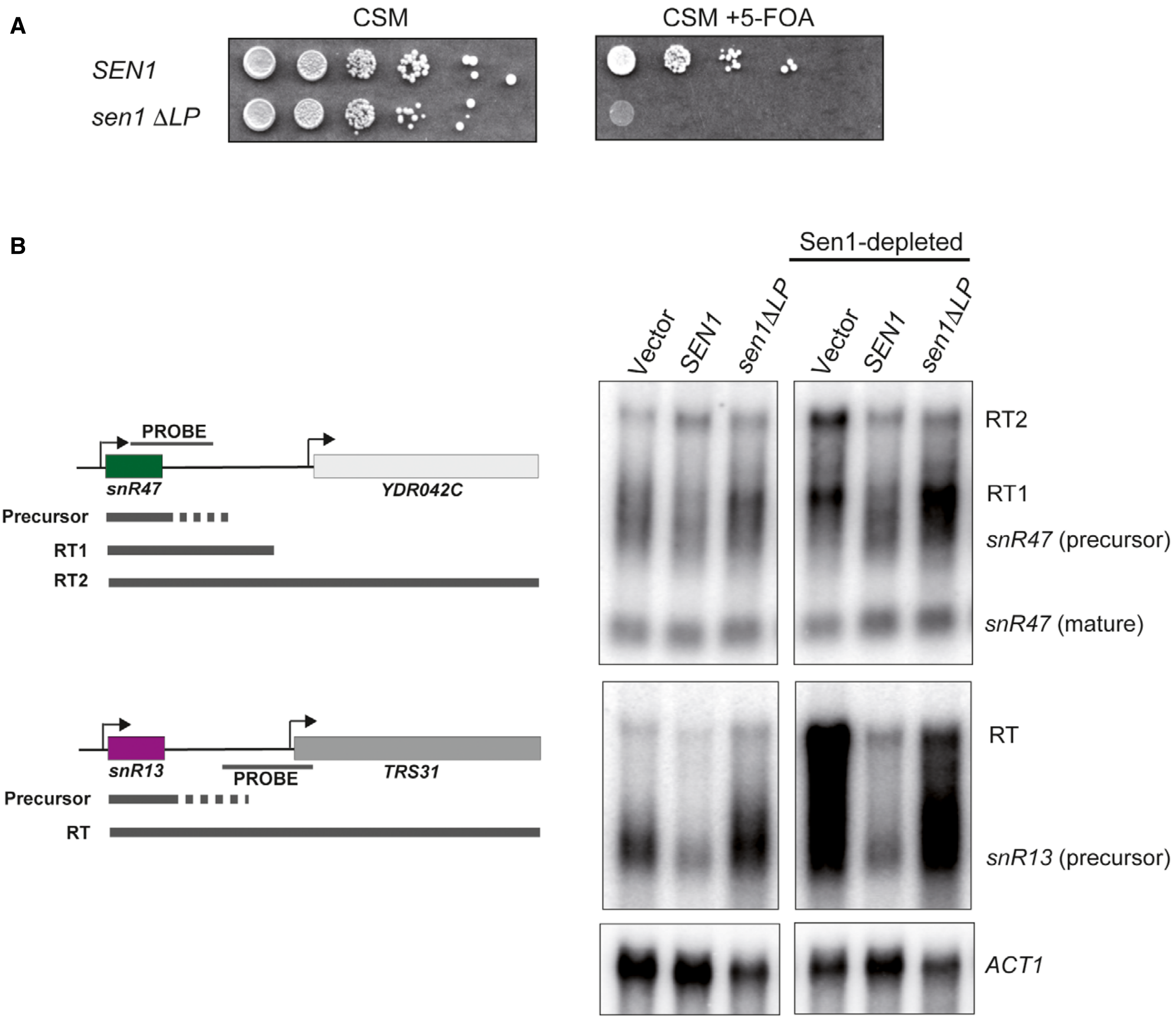

Figure EV4. Analysis of the phenotype of the ΔLP mutant *in vivo*.

A A Sen1 variant harboring the ΔLP cannot support cell viability. A Δ*sen1* strain (YDL2767) covered by an *URA3*-containing plasmid (pFL38) expressing wild-type (wt) Sen1 was transformed with a *TRP1*-plasmid (pFL39) carrying either the wt or a ΔLP version of *SEN1*. After over-night growth in non-selective medium, cells initially harboring both plasmids were plated on minimal medium (CSM) containing 5-fluoroorotic acid (5-FOA) to select for cells that have lost the *URA3* plasmid (and can therefore survive thanks to the *TRP1* plasmid-borne *SEN1* copy). The absence of cells growing in 5-FOA and containing the *TRP1* plasmid expressing Sen1 ΔLP indicates that the ΔLP deletion is lethal.

B The Sen1ΔLP mutant is strongly defective in transcription termination *in vivo*. Northern blot analyses of two well-characterized NNS-targets, snR47 and snR13, in a Sen1-AID (auxin-induced degron, Nishimura *et al*, 2009) strain carrying a plasmid (pFL39) expressing either the wt or a ΔLP version of *SEN1*. A strain harboring an empty vector was included as a positive control for termination defects. To detect the primary products of NNS-dependent termination that are processed/degraded by the exosome, the strain was also deleted in the exonuclease *RRP6*. Sen1-AID was depleted for 1 h by the addition of 100 μM indole-3-acetic acid (a natural auxin) to monitor the capacity of the plasmid-borne versions of *SEN1* to induce transcription termination. The strong accumulation of longer RNA species in the *sen1ΔLP* mutant compared to the wt is indicative of major termination defects. Under non-depletion conditions, the strain harboring the mutant protein exhibits a dominant-negative phenotype (partial termination defects), indicating that Sen1ΔLP has similar expression levels compared to the endogenous Sen1. The *ACT1* transcript is used as a loading control.

 © 2017 The Authors

```
Sen1_S.cerevisiae    1095 AEL-AKQE--LEHMRKRLNVDMNPLYEIILQWDYTRNSEYPDDEPIGN-----YSDVKD 1145
SETX_H.sapiens       1680 KYFPSSSP--VNILL-SSQSVSDTFVKEVLKWKYEMFLNFGQCGPPASLCQSISRPVPV 1735
Upf1_S.cerevisiae     176 SWV-AEQPTEEEKLKARL-ITPSQISKLEAKWRSNKDATINDIDAPEE--QEAIPPLLL 230
IGHMBP2_H.sapiens         -----------------------------------------------------------

Sen1_S.cerevisiae    1146 FFNSPADYQKVMKPLLLLESWQGL----CSSRD--RE-DYKPFSIIVGNRTAVSDFYDV 1197
SETX_H.sapiens       1736 RFHNYGDYFNVFFPLMVLNTFETVAQEWLNSPN--RENFYQ---L--QVRKFPADYIKY 1787
Upf1_S.cerevisiae     231 RYQDAYEYQRSYGPLIKLEADYDKQL--KESQA--LEHISVSWSLALNNRHLASFTLST 285
IGHMBP2_H.sapiens       1 ------------MASAAVESFVTKQLDLLELERDAEVEERRSWQENISLKELQSRG--- 44

Sen1_S.cerevisiae    1198 YASVAK-------QVIQDCGISESD-LIVMAYLPDFRPDK------RLSSDD-----FK 1237
SETX_H.sapiens       1788 WEFA---------VYLEECELAKQL-YPKENDLVFLAPERINEEKKDTERND-----IQ 1831
Upf1_S.cerevisiae     286 FESNELKVAIGDEMILWYSGMQHPD-WEGRGYIVRLP-------------NS-----FQ 325
IGHMBP2_H.sapiens      45 -------------VCLLKLQVSSQRTGLYGRLLVTFEPRRYGSAA-ALPSNSFTSGDIV 89

Sen1_S.cerevisiae    1238 ---K-------AQHTCLAKVRTLKNTKGGNVDVTLRIHRNHSFSKFLTLRSEIYCVKVM 1286
SETX_H.sapiens       1832 DLHE-------YHSGYVHKFRRTSVMRNGKTECYLSIQTQENFPANL--NELVNCIVIS 1881
Upf1_S.cerevisiae     326 ---D-------T---FTLELKPSKTP--------PPTHLTTGFTAEFIWKGTSYDRMQD 363
IGHMBP2_H.sapiens      90 GLYDAANEGSQLATGILTRVTQ--------KSVTVAFDESHDFQLSLDRENSYRLLKLA 140

Sen1_S.cerevisiae    1287 QMTTIEREYSTLEGLEYYDLVGQILQ-AK--P-SPPVNVDAA--------EIETVKK-S 1332
SETX_H.sapiens       1882 SLVTTQRKLKAM------SLLGSRNQLAR--AVLNPNPMDFCTKDLLTTTSERIIAY-L 1931
Upf1_S.cerevisiae     364 ALKKFAIDKKSISGYLYYKILGHQVV-DI--SFDVPLP------------KEFSIPN-F 406
IGHMBP2_H.sapiens     141 NDVTYRRLKKALIALKKYHSGPASSLIEVLFGRSAPSPAS----------EIHPLTFFN 189

Sen1_S.cerevisiae    1333 YKLNTSQAE-------AIVNSVSKEGFSLIQGPPGTGKTKTILGIIGYFLSTKNASSSN 1384
SETX_H.sapiens       1932 RDFNEDQKKAIETAYAMVKHSPSVAKICLIHGPPGTGKSKTIVGLLYRLLTENQRKG-- 1988
Upf1_S.cerevisiae     407 AQLNSSQS--------NAVSHVLQRPLSLIQGPPGTGKTVTSATIVY-HL--------- 447
IGHMBP2_H.sapiens     190 TCLDTSQKE-------AVLFALSQKELAIIHGPPGTGKTTTVVEIILQAV--------- 232

Sen1_S.cerevisiae    1385 VIKVPLEKNSSNTEQLLKKQKILICAPSNAAVDEICLRLKSGVYDKQ-------GHQFK 1436
SETX_H.sapiens       1989 -------HSDENSNAKIKQNRVLVCAPSNAAVDELMKKIILEFKEKCKDKKNPLGNCGD 2040
Upf1_S.cerevisiae     448 --------------SKIHKDRILVCAPSNVAVDHLAAKLRDLGLK-------------- 478
IGHMBP2_H.sapiens     233 ----------------KQGLKVLCCAPSNIAVDNLVERLALCKQR-------------- 261

Sen1_S.cerevisiae    1437 PQLVRVGR--SDVVNVAIKDLTLEELVDKRIGERNYEI----RTDP-ELERKFNNAVTK 1488
SETX_H.sapiens       2041 INLVRLGPE--KSINSEVLKFSLDSQVNHRMKKELPSHVQAMHKRKEFLDYQLDELSRQ 2097
Upf1_S.cerevisiae     479 --VVRLTAKSREDVESSVSNLALHNLVGRG----------------------------- 506
IGHMBP2_H.sapiens     262 --ILRLGHPAR--LLESIQQHSLDAVLARSDSA---QIVADIRKDI-------DQVFVK 306

Sen1_S.cerevisiae    1489 RRELRGKLDSESGNPESPMSTEDISKLQLKIRELSKIINELGRDRDEMREKNSVNYRNR 1547
SETX_H.sapiens       2098 RALCRGGRE--------------I-----QRQELDENISKVSKERQE----LASKIKEV 2133
Upf1_S.cerevisiae     507 ---AKGEL-----------------------KNLLKLKDEVGEL-------SASDTKRF 532
IGHMBP2_H.sapiens     307 NKKTQDK------------------------REKSNFRNEIKLLRKELKER-------- 333

Sen1_S.cerevisiae    1548 DLDRRNAQAHILAVSDIICSTLSGSAHDVLA-----TMGIKFDTVIIDEACQCTELSSI 1601
SETX_H.sapiens       2134 QGRPQKTQSIIILESHIICCTLSTSGGLLLESAFRGQGGVPFSCVIVDEAGQSCEIETL 2192
Upf1_S.cerevisiae     533 VKLVRKTEAEILNKADVVCCTCVGAGDKR--------LDTKFRTVLIDESTQASEPECL 583
IGHMBP2_H.sapiens     334 ---EEAAMLESLTSANVVLATNTG---ASADGPLKLLPESYFDVVVIDECAQALEASC  386

Sen1_S.cerevisiae    1602 IPLRYGGKRCIMVGDPNQLPPTVLSGAASNFKYNQSLFVRME-KN-----------SSP 1648
SETX_H.sapiens       2193 TPLIHRCNKLILVGDPKQLPPTVISMKAQEYGYDQSMMARFCRLLEENVEHNMISRLPI 2251
Upf1_S.cerevisiae     584 IPIVKGAKQVILVGDHQQLGPVILERKAADAGLKQSLFERLISLG-----------HVP 631
IGHMBP2_H.sapiens     387 IPLLK-ARKCILAGDHKQLPPTTVSHKAALAGLSLSLMERLAEEYGA---------RVV 435

Sen1_S.cerevisiae    1649 YLLDVQYRMHPSISKFPSSEFYQGRLKDGPGMDIL----NKRPWHQLEPLAPYKFFDI- 1702
SETX_H.sapiens       2252 LQLTVQYRMHPDICLFPSNYVYNRNLKTNRQTEAIR---CSSDW----PFQPYLVFDV- 2302
Upf1_S.cerevisiae     632 IRLEVQYRMNPYLSEFPSNMFYEGSLQNGVTIEQRTVPNSKFPWPIRG--IPMMFW-A- 686
IGHMBP2_H.sapiens     436 RTLTVQYRMHQAIMRWASDTMYLGQLTAHSSVARHLLRDLPGVAATEETGVPLLLVDTA 494

Sen1_S.cerevisiae    1703 ISGRQEQ-NAKTMSYTNMEEIRVAIELVDYLFRKFDNKIDFTGKIGIISPYREQMQKMR 1760
SETX_H.sapiens       2303 GDGSER---RDNDSYINVQEIKLVMEIIKLIKDK--RKDVSFRNIGIITHYKAQKTMIQ 2356
Upf1_S.cerevisiae     687 NYGREEI-SANGTSFLNRIEAMNCERIITKLFRD----GVKPEQIGVITPYEGQRAYIL 740
IGHMBP2_H.sapiens     495 GCGLFELEEEDEQSKGNPGEVRLVSLHIQALV----DAGVPARDIAVVSPYNLQVDLLR 549

Sen1_S.cerevisiae    1761 KEF--ARYFGGMINKSIDFNTIDGFQGQEKEIILISCVRADDTKSSVGFLKDFRRMNVA 1817
SETX_H.sapiens       2357 KDLD--KEFD--RKGPAEVDTVDAFQGRQKDCVIVTCVRANSIQGSIGFLASLQRLNVT 2411
Upf1_S.cerevisiae     741 QYMQMNGSLDKDLYIKVEVASVDAFQGREKDYIILSCVRANEQ-QAIGFLRDPRRLNVG 798
IGHMBP2_H.sapiens     550 QSLV-------HRHPELEIKSVDGFQGREKEAVILSFVRSNRK-GEVGFLAEDRRINVA 600

Sen1_S.cerevisiae    1818 LTRAKTSIWVLGHQRSLAKSKLWRDLIEDAKDRSCLAYACS-------GFLD------- 1862
SETX_H.sapiens       2412 ITRAKYSLFILGHLRTLMENQHWNQLIQDAQKRGAIIKTCD-------KNYRHDAVKIL 2463
Upf1_S.cerevisiae     799 LTRAKYGLVILGNPRSLARNTLWNHLLIHFREKGCLVEGTL-------DNLQLCTVQLV 850
IGHMBP2_H.sapiens     601 VTRARRHVAVICDSRTVNNHAFLKTLVEYFTQHGEVRTAFEYLDDIVPENYSHENSQGS 659

Sen1_S.cerevisiae    1863 -PRNNRA-QSILRKFNVPVPSEQEDDYKLPMEY-----ITQG-----PDEVKSN      1904
SETX_H.sapiens       2464 KLKPVLQ-----RSLTHPPTIAPEGSR--P----------QG-----GL-----      2490
Upf1_S.cerevisiae     851 RPQPRKTERPMNAQFNVESE--M-GDFPKFQDF-----DAQS-----MVSFSGQ      891
IGHMBP2_H.sapiens     660 SHAA--------TKPQGPATSTRTGSQRQEGGQEAAAPARQGRKKPAGKSLASE      705
```

Figure EV5. Multiple sequence alignment of the helicase domain of Upf1-like helicases.
The multiple alignment was done using Clustal Omega, and the conservation was calculated using BLOSUM62 and is shown in purple.
