# Supplementary figures and images for: Sen1 has unique structural features grafted on the architecture of the Upf1‐like helicase family
Source: EMBO J. 2017 Apr 13;36(11):1590–604. doi: 10.15252/embj.201696174 (PMC5452015; doi:10.15252/embj.201696174)

Figure 1A left

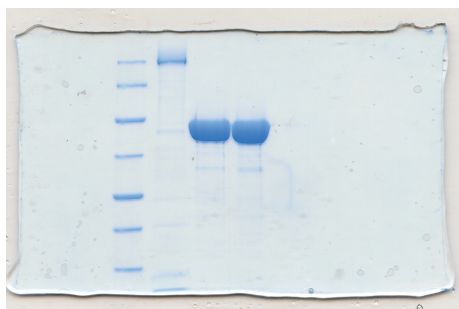

Figure 1B

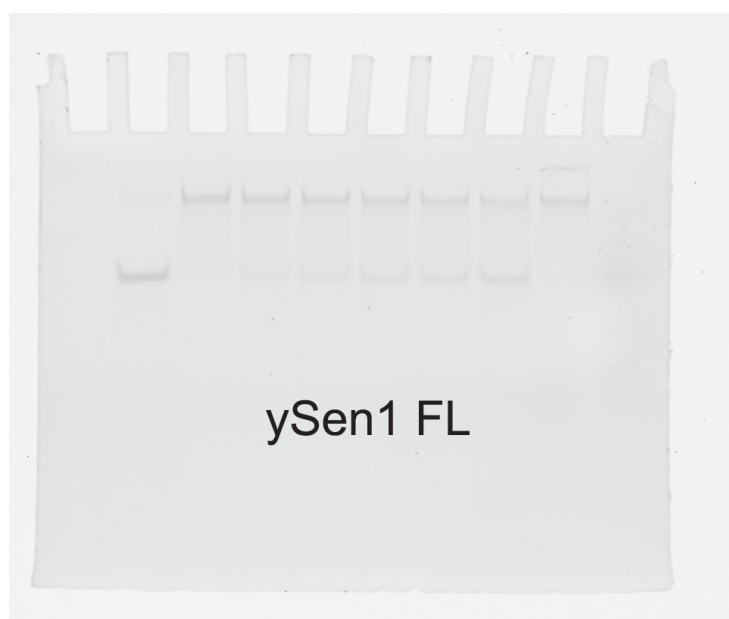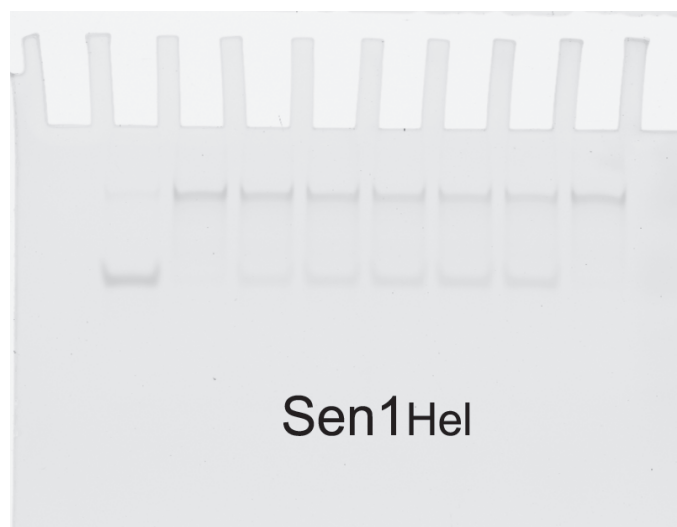

Figure 1C

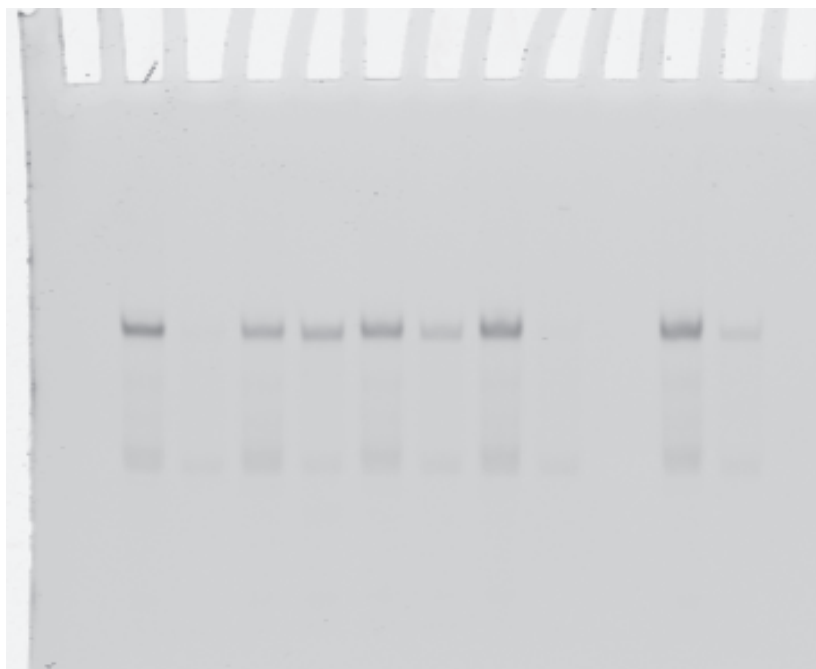

Supplement: Supplementary file 4 — Source Data for Figure 1 [file EMBJ-36-1590-s003.zip › Figure_1_source_data/Figure_1_gels.pdf]

Figure 4B left

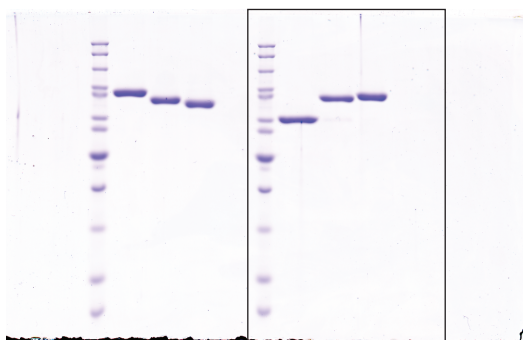

Figure 4B right

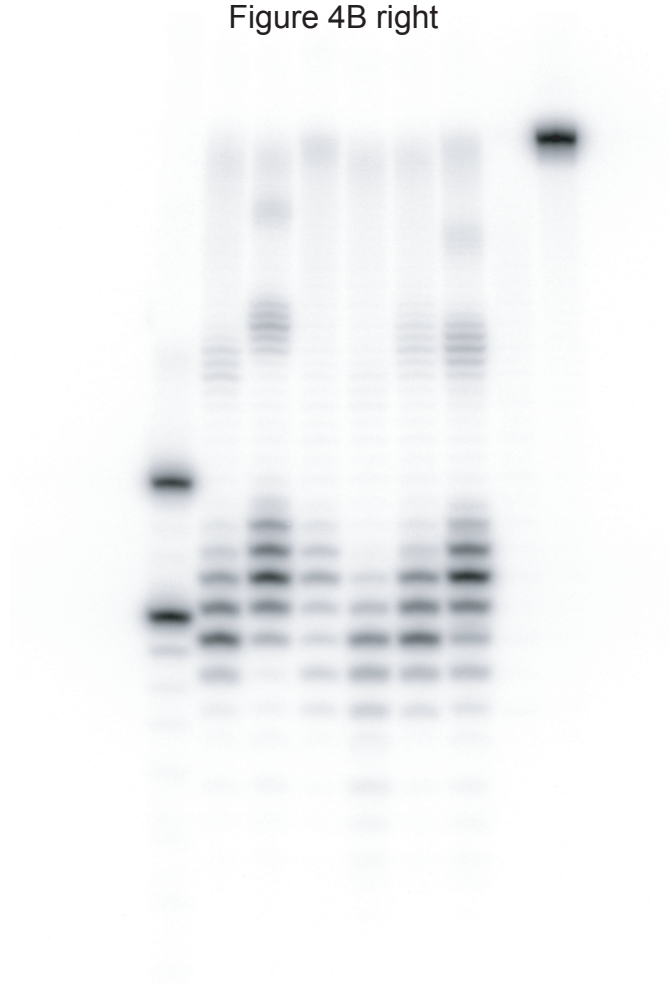

Supplement: Supplementary file 5 — Source Data for Figure 4 [file EMBJ-36-1590-s004.pdf]

Figure 5B left

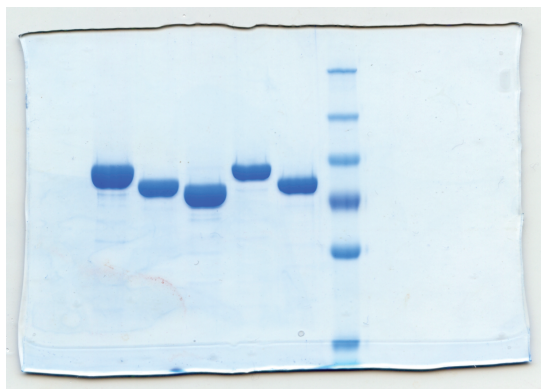

Figure 5B right

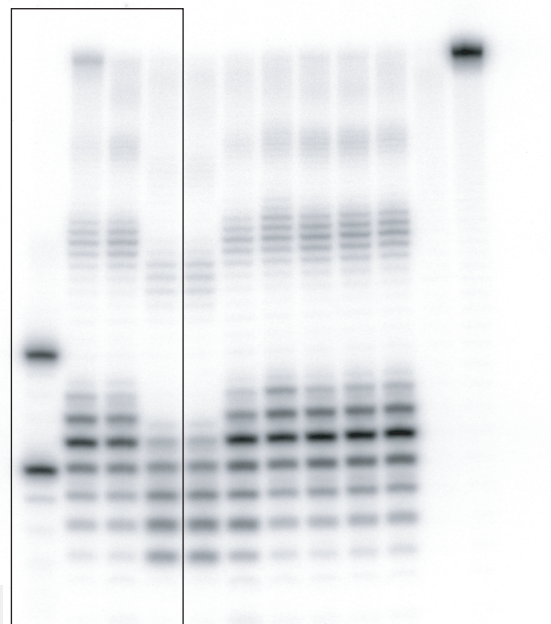

Figure 5D exemplary gels

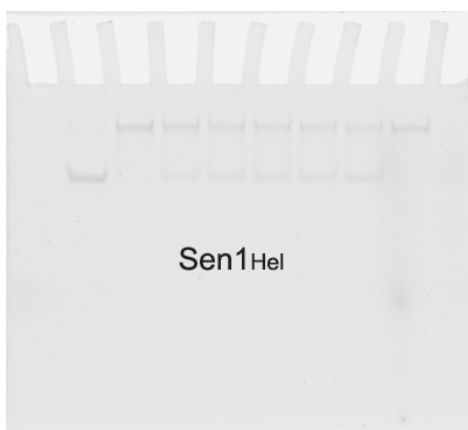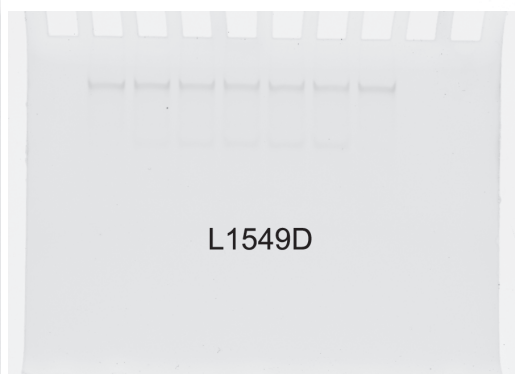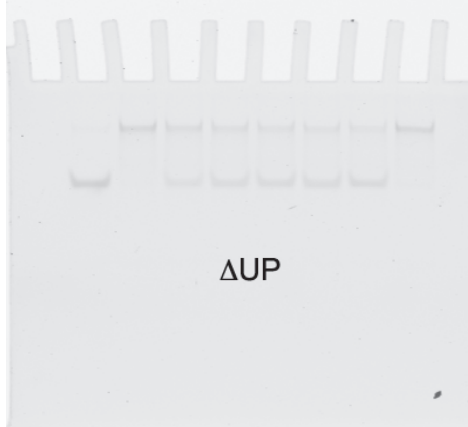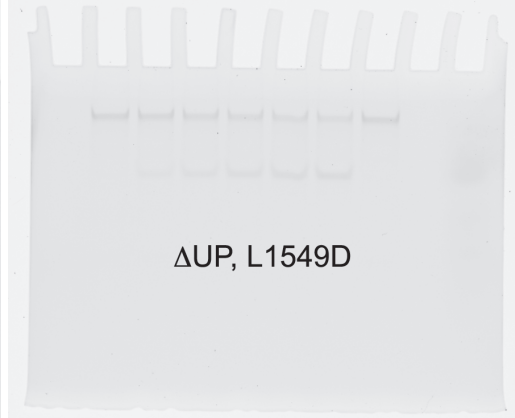

Figure 5E left

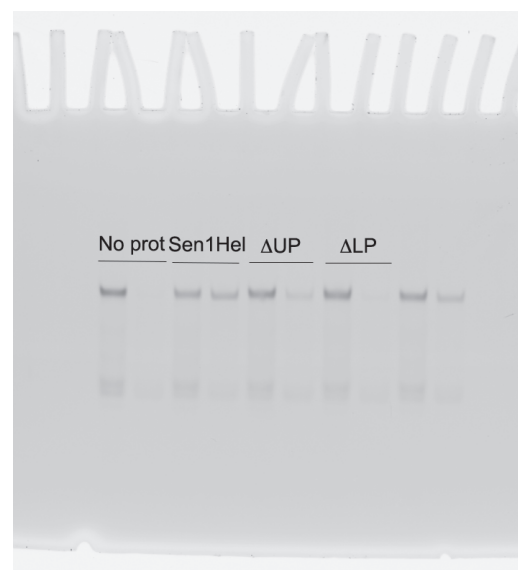

Figure 5E right

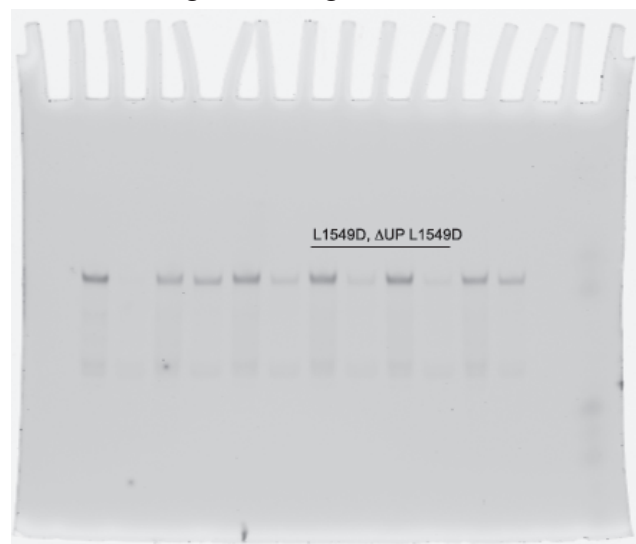

Supplement: Supplementary file 6 — Source Data for Figure 5 [file EMBJ-36-1590-s005.zip › Fig5_Source_Data/Figure_5_gels.pdf]

Figure 6D exemplary gels

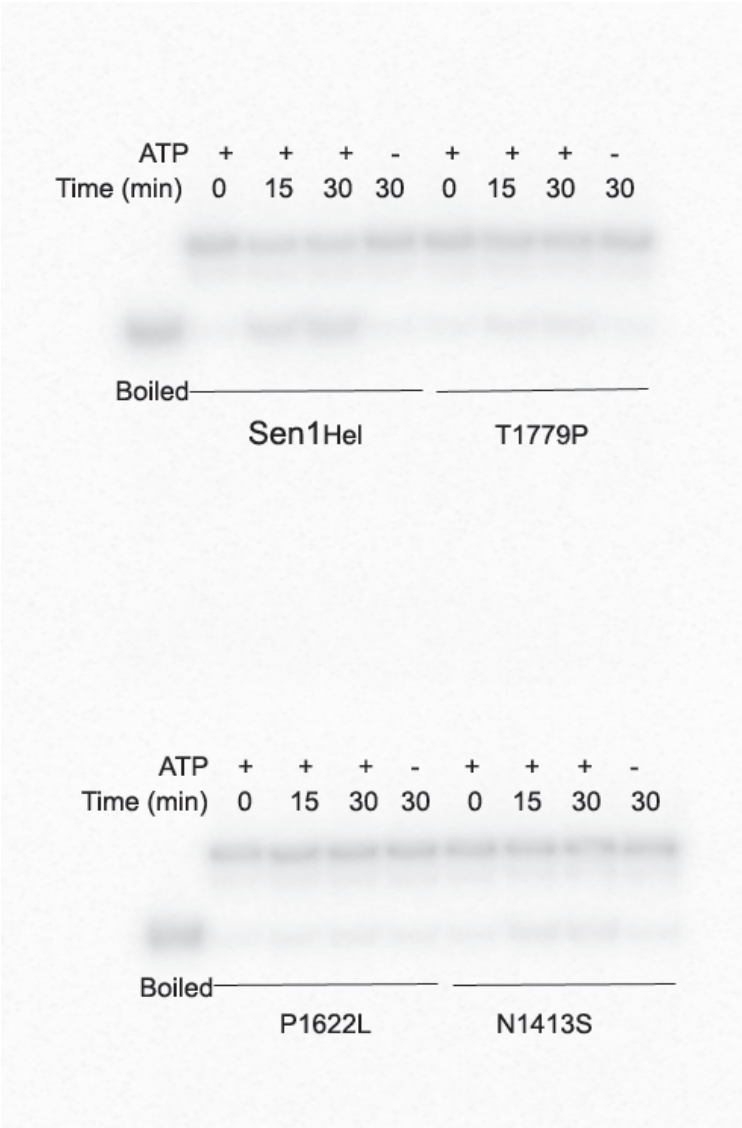

Figure 6E exemplary gels

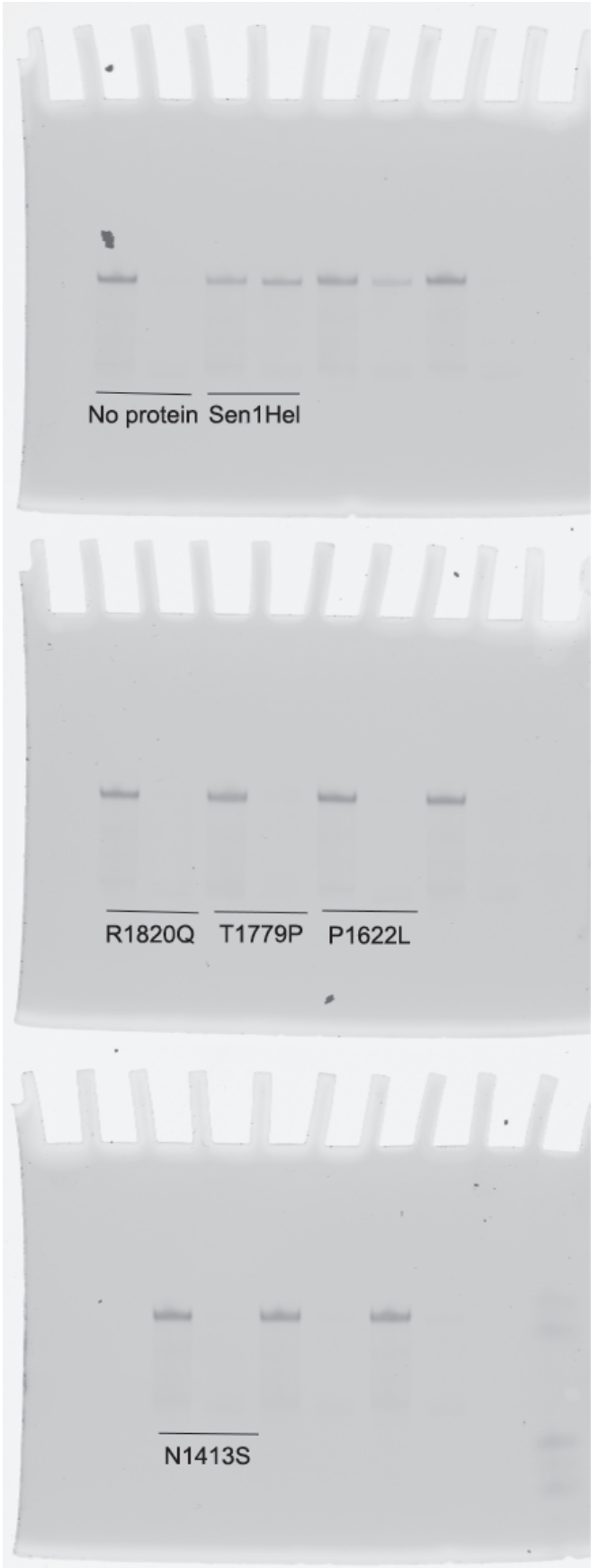

Supplement: Supplementary file 7 — Source Data for Figure 6 [file EMBJ-36-1590-s006.zip › Figure_6_source_data/Figure_6_gels_.pdf]
